# Experiences of sexual and reproductive health screening and counseling in the clinical setting among adolescents and young adults with rheumatic disease

**DOI:** 10.1186/s12969-025-01056-9

**Published:** 2025-01-20

**Authors:** Brittany Huynh, Mary A. Ott, Stacey E. Tarvin

**Affiliations:** 1 Division of Pediatric Rheumatology, Dept. of Pediatrics, Indiana University School of Medicine, 1120 West Michigan St. CL200, Indianapolis, IN 46202 USA; 2 Division of Adolescent Medicine, Dept. of Pediatrics, Indiana University School of Medicine, Indianapolis, IN USA; 3 Department of Global Health and Health Systems Design and Department of Pediatrics, Arnhold Institute for Global Health, Icahn School of Medicine at Mount Sinai, NY, NY USA

**Keywords:** Adolescents, Rheumatic disease, Teratogen, Contraception, Emergency contraception, Long-acting reversible contraception, Reproductive health

## Abstract

**Background:**

Our objective was to describe differences among adolescents and young adults (AYAs) with rheumatic disease using teratogens compared to non-users in receipt of sexual and reproductive health (SRH) counseling, teratogenicity knowledge, perceived importance of SRH topics, and preferences around counseling.

**Methods:**

AYAs ages 14–23 years and assigned female at birth were recruited from pediatric rheumatology clinics at a Midwest tertiary care program. Participants completed a one-time online survey assessing SRH.

**Results:**

One-hundred eight participants completed the survey, representing a range of rheumatic diseases. 24% reported ever having sex. 36% used a teratogen. Rates of screening and counseling regarding SRH topics were low. Notably, pregnancy prevention and emergency contraception (EC) counseling by rheumatologists were uncommon and not associated with teratogen use or sexual activity. Among AYAs on teratogens, only half reported screening for sexual activity or counseling on teratogenicity or pregnancy prevention. Gaps in pregnancy prevention and EC counseling remained even when accounting for counseling by other providers. Knowledge of medication teratogenicity was also low. AYAs reported SRH topics of high importance, and many reported recent concerns. They preferred to receive information from their rheumatologist, and most agreed it is important to talk to their rheumatologist regarding these topics.

**Conclusions:**

AYAs with rheumatic disease report low levels of SRH screening and counseling by their rheumatologist yet report these topics are important and want to discuss them. Gaps in teratogenicity knowledge were identified. This study identifies a need for improved communication with AYAs regarding their SRH.

Adolescent and young adults (AYAs) with rheumatic disease are often treated with teratogenic medications, such as methotrexate, mycophenolate, and cyclophosphamide, increasing pregnancy-related risks. The American College of Rheumatology’s (ACR) guideline for the management of reproductive health in rheumatic and musculoskeletal diseases acknowledges these risks and recommends that rheumatologists discuss contraception and pregnancy plans with all reproductive-age women at an initial or early visit and periodically during follow up [1]. When initiating a potentially teratogenic medication, baseline and periodic sexual and reproductive health (SRH) counseling should include the efficacy and safety of contraception, including emergency contraception [1]. SRH counseling is particularly important for AYAs living in states where access to contraception, abortion, and other components of SRH care is restricted [2, 3]. 

Communication with subspecialty providers regarding SRH is important to individuals with rheumatic disease. Adult women with rheumatic disease report a strong desire for rheumatologists to have a prominent role in SRH care and counseling, and AYAs with rheumatic disease report wanting rheumatologists to be more knowledgeable and active in counseling on SRH topics [4, 5]. For AYAs, this preference for SRH counseling from specialty providers crosses disease categories. For example, AYAs with cystic fibrosis want to discuss SRH topics with their subspecialty providers, and adults with epilepsy and congenital heart disease report viewing their subspecialist as their primary source of SRH information, desiring that this counseling starts in adolescence [6–9]. Concerns around contraception and pregnancy are especially relevant during adolescence and young adulthood. This is a time many young people initiate sexual activity, including those with chronic health conditions, making SRH counseling vital [10]. 

In order to understand the experiences of AYAs with rheumatic disease with SRH screening and counseling, we surveyed AYAs receiving care in pediatric rheumatology clinics in a Midwestern state. The objective of this analysis was to describe AYAs’ experiences of SRH screening and counseling by rheumatology providers, and to describe differences between AYAs using teratogens and non-teratogenic medication users. These experiences included receipt of SRH counseling, teratogenicity knowledge, perceived importance of knowledge on SRH topics, and preferences of AYAs around SRH counseling.

## Patients and methods

### Patients

AYAs between the ages of 14–23 years old, assigned female at birth, and diagnosed with any long-term rheumatic conditions were recruited from a large midwestern tertiary care center and associated outreach clinics between October 2020 and July 2022. This pediatric rheumatology division is the only Indiana-based division providing rheumatology care in the state. AYAs who were followed for pain amplification syndromes, hypermobility, reactive arthritis, or drug-induced lupus, were non-English speaking, or were wards of the state were excluded.

### Procedures

Participants provided their own consent and then completed a one-time electronic survey. Participants also provided permission to extract medical data from their electronic health record. Participants not meeting inclusion criteria (*n* = 5) or missing data on outcome measures (*n* = 1) were excluded. Following completion of the survey, participants received a $15 gift card as compensation for their time. This study was approved by the Indiana University institutional review board with a waiver of parental permission.

### Measures

#### Demographics

Participants were asked to self-report their age, race, and ethnicity.

#### Current teratogen use

Teratogen use was extracted from the electronic medical record from their medication list and review of rheumatology documentation. The 2020 American College of Rheumatology’s (ACR) reproductive health guidelines were used to define teratogenicity of medications [1]. We included common teratogens used in rheumatic disease, including cyclophosphamide, leflunomide, methotrexate, mycophenolate, and angiotensin converting enzyme (ACE) inhibitors. Following release of the ACR guidelines, the FDA released guidance that non-steroidal anti-inflammatory drugs (NSAIDs) should be avoided starting in week 20 of pregnancy [11]. As there is no clear consensus against NSAID use prior to 20 weeks, NSAIDs were excluded from this analysis.

#### Reproductive health practices, knowledge, screening, and counseling

Surveys assessed sexual and reproductive health practices (e.g. sexual experience, birth control use, pregnancy), prior screening and counseling by rheumatologists and by other medical providers in the following SRH areas: sexual activity, teratogenicity, pregnancy prevention, and emergency contraception (e.g. “Has a medical provider ever talked to you about…”), and knowledge on teratogenicity (e.g. “The medicine I am on for my rheumatic disease could be bad for my baby if I were to become pregnant.”). Survey questions are included in a supplemental table.

#### Disease-associated factors

Participants were asked to self-report their disease severity on a scale of 0–10, with 10 being most severe. Primary rheumatic disease was extracted from the electronic medical record as was past teratogen use.

#### Reproductive health importance and preferences

Importance of knowledge on the effects of medications on pregnancy, birth control, and emergency contraception for AYAs with rheumatic disease were rated on a 5 point Likert scale, as was concern regarding these topics in the 3 months prior to the survey. Each item was assessed individually (e.g. “How important is it for people your age (14–23 years old) with a rheumatic disease to know about (topic)?”). Participants were additionally asked their preferred method of receiving information on these topics and importance of discussing SRH topics with their rheumatologist.

### Analysis

Knowledge and counseling questions were largely categorized into trichotomous fields (true/false/unsure or yes/no/unsure). Responses to these questions were dichotomized into true/(false or unsure) and yes/(no or unsure) for analysis. Descriptive statistics are presented as percentages, means, and standard deviations. Differences between teratogen users and non-users in teratogenicity knowledge and self-reported receipt of SRH counseling from pediatric rheumatologists and from any provider were evaluated using χ^2^. Effects of SRH practices and disease-associated factors on SRH counseling categories were evaluated using χ^2^ and logistic regression as appropriate. Differences between groups were considered statistically significant at p-level of ≤ 0.05.

We conducted a multivariable logistic regression for counseling experiences in each of the screening and counseling topics. Factors significant on bivariate analysis at *p* < 0.10 in any category were included in multivariable analysis. Counseling on emergency contraception was excluded from multivariate analysis as only 6 participants reported this counseling from a pediatric rheumatologist. Data were analyzed using SPSS version 29.0.1.0.

## Results

### Participant characteristics (table 1)

Participants (*N* = 108) were on average 16.7 years (SD = 2.0 years) at the time of survey completion with no difference in age between teratogen users and non-users (*p* = 0.405). 76% of participants were White, 10% Black, 8% Latinx, 3% Asian, and 1% American Indian/Alaska Native. A range of rheumatic diseases were represented, with the most common diagnosis being juvenile idiopathic arthritis (JIA, 52%) followed by systemic lupus erythematosus (SLE, 16%). Over one third (36%) were currently prescribed a teratogen at the time of the survey.

### Sexual and reproductive health practices (table 1)

Six (15%) teratogen users reported any sexual experience and 5 of those were currently sexually active; 20 (29%) of teratogen nonusers reported sexual experience and 10 of those were currently sexually active.

Two (5%) teratogen users reported use of long-acting reversible contraceptive (LARC) methods at the time of the survey, including the implant or intrauterine device (IUD), and 6 (15%) reported use of another effective hormonal contraceptive method, including combined oral contraceptive pills, birth control patch, vaginal ring, depot medroxyprogesterone acetate (DMPA) injection, and progestin-only pills.

Among sexually experienced teratogen users, one (17%) reported current use of a LARC (implant), 3 (50%) reported current use of non-LARC effective hormonal contraceptive method (combined oral contraceptive pills), and 2 (33%) reported current use of condoms alone without LARC of effective hormonal contraceptive method.

Three (4%) teratogen non-users reported current use of LARC methods, and 20 (29%) reported current use of another effective hormonal contraceptive.

**Table 1:** Participant characteristics, *N* = 108

				Teratogen		No Teratogen^d^	
*Variable*		All		(*n* = 39)		(*n* = 69)	
Age (years)				Mean (SD)			
	Mean	16.7 (2.0)		16.5 (1.8)		16.9 (2.2)	
				n (Percent)			
*Race/Ethnicity*							
	White	82 (76)		26 (66)		56 (81)	
	Black	11 (10)		5 (13)		6 (9)	
	Latinx	9 (8)		5 (13)		4 (6)	
	Asian	3 (3)		2 (5)		1 (1)	
	American Indian/Alaska Native	1 (1)		1 (3)		0	
	Prefer not to answer	2 (2)		0		2 (3)	
*Rheumatic diagnosis*							
	Juvenile idiopathic arthritis	56 (52)		19 (49)		37 (54)	
	Systemic lupus erythematosus	17 (16)		8 (20)		9 (13)	
	Primary Raynaud’s	6 (6)		1 (3)		5 (7)	
	Juvenile dermatomyositis	5 (5)		2 (5)		3 (4)	
	Scleroderma	5 (5)		3 (8)		2 (3)	
	Vasculitis	5 (5)		1 (3)		4 (6)	
	Idiopathic uveitis	4 (4)		2 (5)		2 (3)	
	MCTD/UCTD^a^	4 (4)		2 (5)		2 (3)	
	Sjogrens syndrome	3 (3)		0		3 (4)	
	Chronic recurrent multifocal osteomyelitis	2 (2)		1 (3)		1 (1)	
	Antiphospholipid antibody syndrome	1 (1)		0		1 (1)	
*Current Teratogen Use*		39 (36)		-		-	
	Methotrexate	28 (26)		28 (72)		-	
	Mycophenolate	11 (10)		11 (28)		-	
	ACE inhibitor	3 (3)		3 (8)		-	
	Cyclophosphamide	1 (1)		1 (3)		-	
	Leflunomide	1 (1)		1 (3)		-	
*Ever sex*							
	Yes	26 (24)		6 (15)		20 (29)	
	No	77 (71)		31 (80)		46 (67)	
	Prefer not to answer	5 (5)		2 (5)		3 (4)	
Ever vaginal sex		23 (21)		5/6 (83)		18/20 (90)	
Currently sexually active		15 (14)		5/6 (83)		10/20 (50)	
Current LARC use^b^		5 (5)		2 (5)		3 (4)	
Among sexually experienced youth		-		1/6 (17)		2/20 (10)	
Current effective hormonal contraceptive use^c^		26 (24)		6 (15)		20 (29)	
Among sexually experienced youth		-		3/6 (50)		11/20 (55)	
Current condom use without hormonal contraception		5 (5)		4 (10)		1 (1)	
Among sexually experienced youth		-		2/6 (33)		1/20 (5)	
Ever Pregnant							
	Yes	1 (1)					
	Prefer not to answer	1 (1)					

^a^Mixed connective tissue disease/undifferentiated connective tissue disease

^b^Long-acting reversible contraception, including the implant, copper intrauterine devices (IUDs), and hormonal IUDs

^c^Includes combined oral contraceptive pills, birth control patch, vaginal ring, depot medroxyprogesterone acetate (DMPA) injection, and progestin-only pills

^d^No statistically significant differences between groups

### Reproductive health screening and counseling

Overall rates of AYA-reported SRH screening and counseling by pediatric rheumatologists were low. Only 38% of AYAs reported ever being asked about sexual activity by their pediatric rheumatologist (Table 2). Rates were higher, at 54%, among teratogen users. About half of teratogen users reported receiving teratogenicity counseling from their pediatric rheumatologist (51%) compared to 54% receiving counseling by any provider. Rates of pregnancy prevention and emergency contraception counseling by rheumatologists were low among teratogen users (23% vs. 8% respectively), though rates were higher when asked about counseling by any provider (59% vs. 28%). Rates of counseling on these topics were not significantly different between teratogen users and non-users. 28% of teratogen users received no screening or counseling from their pediatric rheumatologist on any SRH topic.

**Table 2:** Participant self-report of reproductive health screening and counseling by healthcare providers

					Current Teratogen Use	
			Overall (*N* = 108)		Teratogen (*n* = 39)	None (*n* = 69)
*Reproductive Health Screening/Counseling (Has provider ever…)*			n (Percent)		n (Percent)	n (Percent)
	Pediatric Rheumatologist:					
		Asked about sexual activity	41 (38)		21 (54)	20 (29)
		Counseled risk of disease or medication on health of fetus	35 (32)		20 (51)	15 (22)**
		Told should not get pregnant due to medication for rheumatic disease	29 (27)		17 (44)	12 (17)**
		Talked about how to prevent pregnancy	18 (17)		9 (23)	9 (13)
		Talked about how to get or use emergency contraception	6 (6)		3 (8)	3 (4)
		Received no screening/counseling	50 (46)		11 (28)	39 (57)**
	Any Provider:					
		Asked about sexual activity	89 (82)		33 (85)	56 (81)
		Counseled risk of disease or medication on health of fetus	38 (35)		21 (54)	17 (25)**
		Told should not get pregnant due to medication for rheumatic disease	30 (28)		17 (44)	13 (19)**
		Talked about how to prevent pregnancy	60 (56)		23 (59)	37 (54)
		Talked about how to get or use emergency contraception	22 (20)		11 (28)	11 (16)
		Received no screening/counseling	12 (11)		5 (13)	7 (10)
*Key Knowledge Assessment*						
	“The medicine I am on for my rheumatic disease could be bad for my baby if I were to become pregnant.”					
		Yes	46 (43)		24 (62)	22 (32)**
		No	9 (8)		3 (8)	6 (9)
		Unsure	53 (49)		12 (31)	41 (59)
	“If I get pregnant, I should immediately stop all my medications.”					
		Yes	22(20)		9 (23)	13 (19)
		No	18 (17)		6 (15)	12 (17)
		Unsure	68 (63)		24 (62)	44 (64)

*p < 0.05

**p < 0.01

In bivariate analysis of predictors of SRH screening and counseling by pediatric rheumatologists (Table 3), sexual activity screening was more likely to be reported in teratogen users compared to non-users (*p* = 0.014) and those with higher self-reported disease severity (*p* = 0.05). Teratogenicity counseling was more likely to be reported by current teratogen users (*p* = 0.003), older AYAs (*p* < 0.001), and sexually experienced AYAs (*p* = 0.023), though not those reporting current sexual activity (*p* = 0.226). Counseling on avoiding pregnancy due to teratogen use was more likely to be reported in teratogen users (*p* = 0.006) and AYAs with higher self-reported disease severity (*p* = 0.038). No factors were associated with higher general pregnancy prevention counseling, though older AYAs were more likely to report counseling on emergency contraception (*p* = 0.047). Neither pregnancy prevention counseling nor emergency contraceptive counseling was associated with current teratogen use (*p* = 0.191, *p* = 0.665 respectively) nor with sexual experience (*p* = 0.892, *p* = 0.341 respectively). Frequency of visits to rheumatology and current contraceptive use were not associated with any types of screening or counseling.

**Table 3:** Bivariate analysis of predictors of screening and counseling topics by rheumatologists among adolescents and young adults

Screening and counseling topics:	Asked about sexual activity		Counseled risk of disease or medication on health of fetus		Told should not get pregnant due to medication for rheumatic disease	
	Yes	No	Yes	No	Yes	No
Predictors:	n (%)/mean (SD)	Chi2/mean (SD)	n (%)/mean (SD)	Chi2/mean (SD)	n (%)/mean (SD)	Chi2/mean (SD)
Age (years)	16.80 (2.03)	16.69 (2.06)	**17.83 (2.31)**	16.21 (1.68)***	16.90 (1.84)	16.67 (2.12)
Sexually-experienced		4.326		7.513*		2.983
Yes	8 (31)	-	**13 (50)**	-	7 (27)	-
No	29 (38)	-	**19 (25)**	-	19 (25)	-
I prefer not to answer	4 (80)	-	**3 (60)**	-	3 (60)	-
Current sexual activity		1.103		2.163		1.791
Yes	6 (40)	-	9 (60)	-	5 (33)	-
No	2 (20)	-	3 (30)	-	1 (10)	-
I prefer not to answer	0	-	0 (0)	-	0 (0)	-
Current teratogen use	**21 (54)**	6.539*	**20 (51)**	9.928**	**17 (44)**	8.707**
Ever teratogen use	28 (39)	0.191	26 (37)	1.679	23 (32)	3.241
Self-reported disease severity	**5.41 (2.21)**	**4.52 (2.30)** ^𝚺^	5.11 (1.86)	4.74 (2.49)	**5.52 (1.75)**	4.62 (2.44)*

	Talked about how to prevent pregnancy		Talked about how to get or use emergency contraception		Received no screening or counseling	
	Yes	No	Yes	No	Yes	No
Predictors:	n (%)/mean (SD)	Chi2/mean (SD)	n (%)/mean (SD)	Chi2/mean (SD)	n (%)/mean (SD)	Chi2/mean (SD)
Age (years)	**17.56 (2.38)**	**16.57 (1.94)** ^𝚺^	**18.33 (2.73)**	16.64 (1.97)*	**16.30 (1.83)**	17.10 (2.15)*
Sexually-experienced		0.228		2.152		**5.064** ^𝚺^
Yes	5 (19)	-	1 (4)	-	**11 (42)**	-
No	12 (16)	-	4 (5)	-	**39 (51)**	-
I prefer not to answer	1 (20)	-	1 (20)	-	**0 (0)**	-
Current sexual activity		**4.167** ^𝚺^		0.694		1.732
Yes	**5 (33)**	-	1 (7)	-	5 (33)	-
No	**0 (0)**	-	0 (0)	-	6 (60)	-
I prefer not to answer	**0 (0)**	-	0 (0)	-	0 (0)	-
Current teratogen use	9 (23)	1.806	3 (8)	0.531	**11(28)**	**8.036** ^𝚺^
Ever teratogen use	14 (20)	1.390	**6 (8)**	**3.311** ^𝚺^	32 (45)	0.125
Self-reported disease severity	4.89 (2.11)	4.86 (2.35)	4.83 (2.93)	4.86 (2.28)	**4.28 (2.44)**	5.36 (2.07)*

^𝚺^*p* < 0.10

*p < 0.05

**p < 0.01

***p < 0.001

### Multivariable analysis of reproductive health counseling by pediatric rheumatologists

Age, sexual experience, current teratogen use, and self-reported disease severity were included in multivariable analysis (Table 4). Teratogen users had 2.7 times greater odds of reporting sexual activity screening (*p* = 0.022), and for every additional point of self-reported disease severity participants had 1.2 times greater odds of reporting screening (*p* = 0.044). Current teratogen users had 7.2 times greater odds of reporting teratogenicity counseling (*p* < 0.001), and odds of reporting counseling increased by 1.7 for each 1 year increase in age (*p* < 0.001). Controlling for other factors, sexual experience did not result in greater odds of reporting teratogenicity counseling. Reported pregnancy prevention counseling was associated with none of these factors.

**Table 4:** Multivariate analysis of predictors of screening and counseling topics by rheumatologists among adolescents and young adults

Screening and counseling topics:	Asked about sexual activity			Counseled risk of disease or medication on health of fetus			Told should not get pregnant due to medication for rheumatic disease			Talked about how to prevent pregnancy			Received no screening or counseling		
	R^2^ = 0.155			R^2^ = 0.359			R^2^ = 0.063			R^2^ = 0.088			R^2^ = 0.239		
*Predictors*:	*OR*	*CI*		*OR*	*CI*		*OR*	*CI*		*OR*	*CI*		*OR*	*CI*	
Age	1.116	(0.904–1.378)		**1.703**	(1.301–2.231)***		1.139	(0.900-1.441)		1.289	(1.000-1.660)		**0.741**	(0.591–0.929)**	
Sexually-experienced	2.058	(0.849–4.990)		0.635	(0.247–1.633)		1.400	(0.556–3.523)		0.975	(0.350–2.712)		0.665	(0.270–1.637)	
Current teratogen use	**2.694**	(1.155–6.285)*		**7.259**	(2.461–21.407)***		**3.713**	(1.479–9.325)**		2.319	(0.789–6.811)		**0.276**	(0.110–0.687)**	
Self-reported disease severity	**1.214**	(1.005–1.466)*		1.122	(0.891–1.411)		1.217	(0.985–1.505)		1.015	(0.796–1.294)		**0.783**	(0.647–0.947)*	

*p < 0.05

**p < 0.01

***p < 0.001

### Teratogenicity knowledge

Only 62% of teratogen users knew their medication could be harmful to a developing fetus (Table 2). Interestingly, 32% of participants who were not taking a teratogen still thought their medication could be harmful. High rates of both groups were unsure of their medication’s effects on a fetus (31% vs. 59%, respectively). Both teratogen users and non-users were largely unsure what to do with their medications if they were to become pregnant (62% vs. 64%), and approximately 20% of both groups reported that they should immediately stop all their medications (23% vs. 19%).

### Preferences related to sexual and reproductive health counseling

AYAs attributed high importance to knowledge on reproductive health topics. Among current teratogen users, 85% reported effects of medication on pregnancy, 87% birth control, and 90% emergency contraception was moderately or extremely important for AYAs with a rheumatic disease to know (Table 5). Rates were similar for non-users, with effects of medication on pregnancy being rated highly by more teratogen non-users than among teratogen users (97%, *p* = 0.025). When accounting for past teratogen use, rates were similar between groups (Table 5). Interestingly, teratogen users who knew the potential harm of their medication to a fetus rated all categories more highly than those without this knowledge, with 100% rating effects of medication on pregnancy as moderately or extremely important (vs. 60% without teratogenicity knowledge, *p* = 0.002), 96% birth control (vs. 73%, *p* = 0.062), and 96% emergency contraception (vs. 80%, *p* = 0.279).

About one third of patients reported a personal concern regarding medication effects and 38% regarding birth control in the three months prior to the survey, with rates higher for teratogen non-users than for teratogen users (NS).

Most AYAs preferred to talk to a healthcare provider about these topics (57%) with their rheumatologist being most preferred (84%). 82% felt it is important to talk with their rheumatologist about SRH topics. Rates were not different between teratogen users and non-users.

**Table 5:** Participant rated importance of reproductive health counseling and preferences regarding education

				Overall				Teratogen				No Teratogen		
Report topic to be moderately or extremely important to know^a^				n (Percent)				n (Percent)				n (Percent)		
	*Among those with current use*:													
		Effects of medication on pregnancy		100 (93%)				**33 (85%)**				67 (97%)*		
		Birth control		92 (85%)				34 (87%)				58 (84%)		
		Emergency contraception		91 (84%)				35 (90%)				56 (81%)		
	*Among those with any past or present use*:													
		Effects of medication on pregnancy						65 (92%)				35 (95%)		
		Birth control						62 (87%)				30 (81%)		
		Emergency contraception						58 (81%)				33 (89%)		
	*Among those with current teratogen use and knowledge of teratogenicity*:^*b*^							*Teratogen + Knowledge*				*Teratogen + No Knowledge*		
		Effects of medication on pregnancy						**24 (100%)**				9 (60%)**		
		Birth control						23 (96%)				11 (73%)		
		Emergency contraception						23 (96%)				12 (80%)		
Report concern about topic in the 3 months prior to survey														
		Effects of medication on pregnancy		34 (32%)				8 (21%)				25 (36%)		
		Birth control		41 (38%)				**10 (26%)**				**30 (44%)** ^𝚺^		
		Emergency contraception		15 (14%)				3 (8%)				11 (16%)		
Preferred method of receiving information on these topics														
		Talk to a healthcare provider^c^		62 (57%)				22 (57%)				40 (58%)		
			Rheumatologist	52/62 (84%)				19 (86%)				33 (83%)		
			Primary care provider	41/62 (66%)				13 (60%)				28 (70%)		
			OB/GYN or Adolescent medicine	36/62 (58%)				10 (46%)				26 (65%)		
			Family planning clinic	5/62 (8%)				1 (5%)				4 (10%)		
		Email		50 (46%)				19 (49%)				31 (45%)		
		Website		42 (39%)				11 (28%)				31 (45%)		
		Webinar/Podcast		7 (6%)				2 (5%)				5 (7%)		
		None		12 (11%)				4 (10%)				8 (12%)		
Report it is important to talk to their rheumatologist about these topics														
		Yes		88 (82%)				34 (87%)				54 (78%)		
		No		5 (5%)				1 (3%)				4 (6%)		
		Unsure		15 (14%)				4 (10%)				11 (16%)		

^a^For people their age with a rheumatic disease; rated 4–5 on a 5 point Likert scale

^b^Selected “True” to the statement: “The medicine I am on for my rheumatic disease could be bad for my baby if I were to become pregnant.”

^c^Select all that apply

^𝚺^*p* < 0.10

*p < 0.05

**p < 0.01

## Discussion

Overall rates of SRH screening and counseling were low for both AYAs using teratogens and non-users. Despite pregnancy-associated medication risks, only half of those on teratogens reported ever being asked by their rheumatologist if they were sexually active. Of particular concern, less than a quarter of participants reported counseling by rheumatologists on pregnancy prevention or emergency contraception, with no significant difference based on teratogen use or sexual experience. These low rates contrast with the high levels of importance placed by AYA participants on contraceptive knowledge. Together these findings indicate that appropriate SRH counseling is not occurring, and the counseling that is provided currently is not focused on those at greatest risk for poor perinatal outcomes.

Our findings are similar to those previously reported among pediatric subspecialists prescribing teratogens, where similar size gaps were found in sexual history documentation and contraceptive counseling (28–32%) [12, 13], and in studies of adults with rheumatic disease on teratogens, where less than 30% reported contraceptive counseling [14]. Provider self-reports from multiple pediatric subspecialties are not much higher than AYA reports, with provider self-reports of sexual activity screening of 20–55% and emergency contraception counseling of 5–9% [15]. Together these findings demonstrate a need for structural and system-level improvements in adult and pediatric subspecialty provider training and for the improvement of SRH screening and counseling for AYAs in subspecialty clinics.

Coupled with the low reported rates of teratogen counseling, participants on teratogens reported low knowledge about the effect of their medications on pregnancy. For example, 38% of teratogen users did not know their medication could harm a fetus if they were to become pregnant. These findings are consistent with studies in adults which demonstrate gaps in SRH knowledge, including potentially detrimental effects of both their chronic disease and medication on pregnancy [16, 17]. Together these findings suggest a high need for multi-level (system, patient, provider) interventions in SRH care for AYAs with rheumatic disease.

Although low, the rate of teratogenicity counseling by our participants was higher than reported rates in studies that relied on chart review in the context of quality improvement (25-32% pre-intervention) [18, 19]. These findings argue against recall accounting for low reported rates of counseling. We also note that rates of teratogenicity counseling were similar for rheumatologists versus any medical provider, indicating this counseling is largely happening by pediatric rheumatologists if at all.

Our findings support the need for improved SRH counseling among AYAs with rheumatic disease treated in pediatric rheumatology clinics. This is in line with quality indicators for adults with inflammatory arthritis and SLE as well as the ACR’s reproductive health guideline, which all recommend teratogenicity counseling when starting a potentially teratogenic medication, discussing contraception, and discussing use of emergency contraception with all patients [1, 20–22]. 

While current guidelines highlight reproductive health care needs for adults with rheumatic disease, sexual activity is often initiated in adolescence, and adolescents with chronic disease are generally sexually active at rates similar to their peers [10, 23]. Additionally, AYAs have the highest rates of unintended pregnancy among any age group, describing 75% of pregnancies in this age range, which risks high disease activity and teratogenic medication use at the time of pregnancy [24]. For AYAs living in states with restrictive laws limiting minor contraceptive access, abortion, and other SRH care, SRH counseling becomes even more critical for AYAs taking teratogens. These AYAs may have fewer reproductive health options to prevent a pregnancy or make choices about a pregnancy. Therefore, it is vital to integrate reproductive health counseling recommendations for this age group into future guidelines. Equipping AYAs with knowledge regarding teratogenicity and contraception allows them to participate in shared decision-making regarding their reproductive health care, applying the principles of reproductive justice, highlighting choice and access, to rheumatology practice [25]. 

In this study, AYAs with rheumatic disease placed high value on knowledge regarding effects of medication on pregnancy, birth control, and emergency contraception, and felt it was important to talk to their rheumatologist about SRH topics. Among those AYAs who preferred to receive this information from a medical provider over written or other materials, their rheumatologist was most preferred, even over primary care, obstetrics and gynecology, or adolescent medicine. This is also supported in the existing literature, with adults and AYAs with rheumatic disease reporting a strong desire for their rheumatologists to have a prominent role in SRH care and counseling, and those with other chronic conditions viewing their subspecialist as their primary source of SRH information [4–9]. This may stem in part from a lack of confidence in the subspecialty knowledge of these other providers, which has been described in qualitative studies in adults and AYAs with rheumatic disease as well as other chronic conditions, though more research is needed to explore these preferences [4, 5, 9, 26]. 

A limitation of this study is a small sample size. Although recruitment occurred across three sites, and ours is the only Indiana-based pediatric rheumatology practice in the state, a larger study is needed to increase statistical power. As the largest study of SRH among AYAs with rheumatic disease to date, our findings serve as a starting point for further evaluation of the SRH needs of AYAs with different types of rheumatic diseases and in different geographic regions of the country.

## Conclusions

Best practice recommends individuals on teratogens are provided with SRH counseling. This study highlights gaps in application of these best practices to adolescents and young adults with rheumatic disease treated in pediatric clinics. We found that AYAs with rheumatic disease report low levels of SRH screening and counseling by their rheumatologist. All AYAs, including those on teratogens, had low rates of pregnancy prevention counseling even when including counseling from non-rheumatology providers. Consistent with low rates of counseling, gaps in knowledge were identified among teratogen users with only 62% recognizing their medication as harmful in pregnancy. Despite these gaps in counseling and knowledge, AYAs attribute high importance to these topics and want to discuss them with their rheumatologist.

The results of this study highlight a high need for multi-level interventions in SRH care for AYAs with rheumatic disease. We suggest the following practical steps in addressing this need:

- Enhanced subspecialty provider training in SRH care of AYAs with rheumatic disease, including counseling on teratogenicity and pregnancy prevention, as these topics may not be addressed by other medical providers.
- Enhanced screening and counseling for AYAs in subspecialty clinics.
- Inclusion of AYAs in future guidelines regarding reproductive health counseling recommendations.
- Development of point of care tools to facilitate shared decision making amongst AYAs and caregivers.

## Data Availability

The datasets used and/or analysed during the current study are available from the corresponding author on reasonable request.

## Abbreviations

ACE: Angiotensin converting enzyme
ACR: American college of rheumatology
AYA: Adolescent and young adult
DMPA: Depot medroxyprogesterone acetate
EC: Emergency contraception
JIA: Juvenile idiopathic arthritis
LARC: Long-acting reversible contraceptive
NSAID: Non-steroidal anti-inflammatory drugs
SLE: Systemic lupus erythematosus
SRH: Sexual and reproductive health
